# Supplementary material for: The ribose methylation enzyme FTSJ1 has a conserved role in neuron morphology and learning performance
Source: Life Sci Alliance. 2023 Jan 31;6(4):e202201877. doi: 10.26508/lsa.202201877 (PMC9889914; doi:10.26508/lsa.202201877)
Supplement: Supplementary file 1 [file LSA-2022-01877_TableS1.docx]

**Table S1.** FTSJ1 loss of function leads to miRNAs deregulation in XLID affected individuals LCLs. A list of the significantly deregulated miRNAs and their log2 fold change, p.values and adjusted p.values between FTSJ1 loss-of-function LCLs and control LCLs.

|  |  | baseMean_mutant | baseMean_wt | log2FoldChange_Mutant_vs_WT | pvalue | padj |
| --- | --- | --- | --- | --- | --- | --- |
| 1 | **hsa-miR-20b-5p** | 3987 | 538 | 2.16 | 1.52E-09 | 1.21E-06 |
| 2 | **hsa-miR-222-3p** | 16116 | 6101 | 1.55 | 1.90E-08 | 6.24E-06 |
| 3 | **hsa-miR-548ax** | 42 | 3 | 3.91 | 2.37E-08 | 6.24E-06 |
| 4 | **hsa-miR-125b-2-3p** | 235 | 1295 | -4.43 | 6.94E-08 | 1.37E-05 |
| 5 | **hsa-miR-221-3p** | 37248 | 12664 | 1.68 | 2.37E-07 | 3.76E-05 |
| 6 | **hsa-miR-335-3p** | 217 | 690 | -2.78 | 6.53E-07 | 7.92E-05 |
| 7 | **hsa-miR-181b-2-3p** | 142 | 44 | 1.82 | 7.00E-07 | 7.92E-05 |
| 8 | **hsa-miR-99a-5p** | 538 | 2186 | -3.61 | 1.36E-06 | 0.0001 |
| 9 | **hsa-miR-10a-5p** | 161396 | 49508 | 2.31 | 2.60E-06 | 0.0002 |
| 10 | **hsa-miR-181b-3p** | 472 | 111 | 2.18 | 6.79E-06 | 0.0005 |
| 11 | **hsa-miR-106a-5p** | 1737 | 547 | 1.10 | 6.94E-06 | 0.0005 |
| 12 | **hsa-miR-181a-2-3p** | 12183 | 4280 | 1.58 | 1.44E-05 | 0.0009 |
| 13 | **hsa-miR-146a-5p** | 256346 | 121011 | 1.15 | 1.54E-05 | 0.0009 |
| 14 | **hsa-miR-4482-3p** | 2 | 58 | -4.97 | 1.57E-05 | 0.0009 |
| 15 | **hsa-miR-125b-5p** | 468 | 1354 | -3.41 | 1.76E-05 | 0.0009 |
| 16 | **hsa-miR-450b-5p** | 235 | 454 | -1.97 | 1.83E-05 | 0.0009 |
| 17 | **hsa-miR-424-3p** | 608 | 750 | -1.63 | 2.55E-05 | 0.0012 |
| 18 | **hsa-miR-363-3p** | 10299 | 2814 | 1.88 | 3.79E-05 | 0.0017 |
| 19 | **hsa-let-7c-5p** | 1044 | 3949 | -2.59 | 4.17E-05 | 0.0017 |
| 20 | **hsa-miR-450a-5p** | 157 | 254 | -1.73 | 5.17E-05 | 0.0020 |
| 21 | **hsa-miR-18b-5p** | 131 | 34 | 1.45 | 8.67E-05 | 0.0033 |
| 22 | **hsa-miR-550a-3p** | 141 | 288 | -1.33 | 9.04E-05 | 0.0033 |
| 23 | **hsa-miR-181a-5p** | 1097695 | 379340 | 1.82 | 9.61E-05 | 0.0033 |
| 24 | **hsa-miR-550b-2-5p** | 94 | 192 | -1.35 | 0.0001 | 0.0044 |
| 25 | **hsa-miR-181a-3p** | 15949 | 4441 | 2.02 | 0.0002 | 0.0051 |
| 26 | **hsa-miR-181b-5p** | 83655 | 32365 | 1.52 | 0.0003 | 0.0100 |
| 27 | **hsa-miR-183-5p** | 391 | 848 | -1.45 | 0.0004 | 0.0112 |
| 28 | **hsa-miR-99a-3p** | 24 | 55 | -3.44 | 0.0005 | 0.0134 |
| 29 | **hsa-miR-135a-5p** | 6 | 80 | -3.81 | 0.0005 | 0.0135 |
| 30 | **hsa-miR-146b-5p** | 70718 | 29704 | 1.41 | 0.0007 | 0.0190 |
| 31 | **hsa-miR-542-5p** | 13 | 19 | -2.65 | 0.0013 | 0.0321 |
| 32 | **hsa-miR-944** | 138 | 833 | -2.53 | 0.0015 | 0.0376 |
| 33 | **hsa-miR-625-5p** | 707 | 1186 | -0.79 | 0.0016 | 0.0395 |
| 34 | **hsa-miR-625-3p** | 723 | 1220 | -0.79 | 0.0018 | 0.0412 |
| 35 | **hsa-miR-4772-5p** | 56 | 26 | 1.77 | 0.0019 | 0.0420 |
| 36 | **hsa-miR-182-5p** | 8091 | 15213 | -1.26 | 0.0022 | 0.0473 |
